# Association study of a genetic variant in the long intergenic noncoding RNA (linc01080) with schizophrenia in Han Chinese

**DOI:** 10.1186/s12888-021-03623-2

**Published:** 2021-12-08

**Authors:** Yi Qi, Yaxue Wei, Fengyan Yu, Qianxing Lin, Jingwen Yin, Jiawu Fu, Susu Xiong, Dong Lv, Zhun Dai, Qian Peng, Ying Wang, Dandan Zhang, Lulu Wang, Xiaoqing Ye, Zhixiong Lin, Juda Lin, Guoda Ma, Keshen Li, Xudong Luo

**Affiliations:** 1 The Marine Biomedical Research Institute, Guangdong Medical University, Zhanjiang, 524023 China; 2 Department of Psychiatry, Affiliated Hospital of Guangdong Medical University, Zhanjiang, 524001 China; 3 Psychiatric and Psychological Clinical Rehabilitation Center, The People’s Hospital of Guangxi Zhuang Autonomous Region, Nanning, 530021 China; 4 The Second Clinical School, Guangdong Medical University, Dongguan, 523808 China; 5 Department of Neurology, Affiliated Hospital of Guangdong Medical University, Zhanjiang, 524001 China; 6 Maternal and Children’s Health Research Institute, Shunde Maternal and Children’s Hospital, Guangdong Medical University, Foshan, 528300 China; 7 Department of Neurology and Stroke Center, The First Affiliated Hospital, Jinan University, Guangzhou, 510630 China; 8 Clinical Neuroscience Institute of Jinan University, Guangzhou, 510630 China

**Keywords:** Schizophrenia, Long intergenic noncoding RNA 01080 (linc01080), SNP rs7990916, Age of onset, Neurocognitive function

## Abstract

**Background:**

Schizophrenia is currently considered to be a polygene-related disease with unknown etiology. This research will verify whether the single nucleotide polymorphism (SNP) of the long intergenic noncoding RNA01080 (linc01080) contributes to the susceptibility and phenotypic heterogeneity of schizophrenia, with a view to providing data support for the prevention and individualized treatment of this disease.

**Method:**

The SNP rs7990916 in linc01080 were genotyped in 1139 schizophrenic and 1039 controls in a Southern Chinese Han population by the improved multiplex ligation detection reaction (imLDR) technique. Meanwhile, we assessed and analyzed the association between this SNP and schizophrenics’ clinical symptoms, and the cognitive function.

**Result:**

There was no significant difference in genotype distribution, allele frequency distribution, gender stratification analysis between the two groups. However, the SNP of rs7990916 was significantly associated with the age of onset in patients with schizophrenia (*P* = 8.22E-07), patients with T allele had earlier onset age compared with CC genotype carriers. In terms of cognitive function, patients with T allele scored lower than CC genotype carriers in the Tower of London score and symbol coding score in the Brief assessment of Cognition (BACS), and the difference was statistically significant (*P* = 0.014, *P* = 0.022, respectively).

**Conclusion:**

Our data show for the first time that linc01080 polymorphism may affect the age of onset and neurocognitive function in patients with schizophrenia.

## Key findings

Linc01080 rs7990916 T allele may be a risk factor affecting the onset of age and cognitive impairment (information processing speed and problem-solving ability) of Han schizophrenic patients in southern China.

## Background

Schizophrenia is a disease with different heterogeneity in clinical symptoms, onset age, cognitive function and brain structure [1], which was once regarded as basing on abnormal neurodevelopment. Various genetic and environmental risk factors can affect early and critical periods of brain development in adolescents, which may eventually lead to the emergence of mental symptoms in adolescence and early adulthood [2]. A study shows that the decline of neural stem cell proliferation level may contribute to the onset of schizophrenia [3].

Long intergenic noncoding RNA (lincRNA) could be defined as an intergenic transcribed fragment that lacks protein-coding capabilities. Structurally, most of lincRNA is mainly concentrated in the nucleus, containing more than 200 nucleotides in length [4], with the characteristics of 59m7G cap and Poly(A) tail, and thus easily regulated by key transcription factors [5]. At the gene level, lincRNAs play a role in gene expression mainly through regulating histone modification, DNA methylation, chromatin remodeling modulated and interacting with transcription factors [6]. Accumulating evidences have shown that lincRNA is linked to neurodevelopment and brain evolution, and also plays a critical role in the regulation of neural stem cells (NSCs) [7]. For examples, LincRNA1230 can limit the junction of WD repeat 5 to the promoter region of neural lineage related genes, thus it could prevent the transformation of mouse neural stem cells into neural progenitor cells [8], LncRNAlinc00115 regulates the self-renewal and proliferation of neural stem cells by activating transforming growth factor-β and linc00152 through activating miR-103a-3p / FEZF axis [9, 10], Linc01198 promotes glioma proliferation by enhancing NEDD4–1-dependent PTEN inhibition [11]. This proliferation and differentiation function of NSCs is obviously related to brain homeostasis, and may even change in mental and nervous system diseases [12].

In a previous study, a genetic variation (rs7990916T > C) was found in linc01080 (also known as lincRNA-Tcon_00021856), with a total length of 148,213 bp and two exons of 130 bp and 627 bp respectively [13]. Further study of the variation found that the distribution of gray matter volume (involving 40 brain regions, including frontal lobe, temporal lobe, parietal lobe and occipital lobe) of different genotypes was statistically different, and the gray matter volume of individuals carrying CC was significantly larger than that of individuals with TT. These findings suggest that this SNP may be related to the cortical development of memory-related brain regions [13]. As far as we know, linc01080 gene polymorphisms have not been studied in schizophrenic patients yet. Based on the fact that the genetic variation of rs9970916 in linc01080 may affect the potential role of neurodevelopment in the pathogenesis of schizophrenia by changing the brain structure, we conducted this study to detect whether this SNP (linc01080 rs7990916) is related to the susceptibility of schizophrenia and the severity of symptoms.

## Materials and methods

### Statement

The Ethics Committee of the Affiliated Hospital of Guangdong Medical University approved the study protocol and obtained written informed consent from all participants included in the study, all methods were performed in accordance with the relevant guidelines and regulations.

### Subjects

The 1139 schizophrenics (male 722, female 417) with the definite diagnosis were selected from the Affiliated Hospital of Guangdong Medical University. The inclusion criteria of all patients followed: (1) Han population in southern China; (2) schizophrenia was diagnosed according to DSM-5, which was conducted by at least two experienced senior psychiatrists. The exclusion criteria included: (1) organic disorders, substance abuse mental disorders, Schizoaffective disorder and mood disorder (2) suffering from serious physical diseases, such as confirmed renal insufficiency, heart disease. The 1039 healthy individuals in the same period were recruited from the Affiliated Hospital of Guangdong Medical University as the control group. The exclusion criteria included: (1) having own or family history of mental illness; (2) having severe physical and / or nervous system diseases; (3) having a long history of substance abuse; (4) having obvious organic lesions on head imaging.

### Symptoms and neurocognitive functions assessment

Schizophrenics’ symptoms were assessed by Positive and Negative Symptom Scale (PANSS), including PANSS total score, positive symptoms, negative symptoms and general psychopathology scale. Since the severity of schizophrenia is easily affected by social background, we include variables such as years of education, course of disease and family history of mental illness, which will make this study more convincing. In addition, the neurocognitive function is assessed by BACS (BACS was designed to be easy to administer and score, and has been used in more than 30 clinical trials for schizophrenia. It is specifically designed to measure cognitive changes associated with treatment and comes in different forms, and its reliability, effectiveness and sensitivity have been demonstrated [14]), in which each items were used to assessed specific cognitive function, including Digit sequencing task (working memory), List learning (verbal fluency), Token motor task (motor speed), Tower of London (reasoning and problem solving), Symbol coding (attention and processing speed), Category instances and Controlled oral word association test (semantic and letter fluency). More details of the BACS assessment please refer to the report of Keefe et al. [14].

### DNA extraction and genotyping

Genomic DNA from EDTA-anticoagulated peripheral blood was extracted using the TIANamp Blood DNA Kit (Tiangen Biotech, Beijing, China). The rs7990916 SNP was genotyped using the improved multiplex ligation detection reaction (iMLDR) method (Genesky Biotechnologies Inc., Shanghai, China), and the detailed experimental steps are as follows: (1) DNA samples were taken 1 μl 1% Agarose electrophoresis for quality check and concentration estimation, and then diluted to the working concentration 5-10 ng/μl according to the estimated concentration. (2) Multiplex PCR reaction was carried out with forward primer 5′-TGTAATGGACCAGTGTGATATCTTGCA-3′ and reverse primer 5′-GCTAATTGTGTAGTGCTGAAGAACACC-3′. PCR condition: the reaction system (20 μl) included 1x HotStarTaq buffer, 3.0 mM Mg2+, 0.3 mM dNTP, 1 U HotStarTaq polymerase (Qiagen Inc.), 1 μl sample DNA and 1 μl multiple PCR primers. (3) Purification of multiple PCR products: 5 U SAP enzyme and 2 U Exonuclease I enzyme were added to 20 μl PCR product, 37 °C warm bath for 1 h, then 75 °C inactivated for 15 min. (4) Ligating reaction system: 10x ligating buffer 1ul, high temperature ligase 0.25ul, 5 ‘ligating primer mixture (1 μ M) 0.4 ul, primer 3’ ligating primer mixture (2 μM) 0.4ul, purified multiple PCR products 2ul, ddH2O6ul mixing. (5) Take 0.5 μl diluted product, mix it with 0.5 μl Liz500 SIZE STANDARD, 9 μl Hi-Di, denatured at 95 °C for 5 min, then put it on the ABI3730XL sequencer. (6) The raw data collected on the ABI3730XL sequencer are analyzed by GeneMapper 4.1 (AppliedBiosystems, USA).

### Statistical analysis

Given the low frequency of the TT genotype in both schizophrenic patients (1.9%) and controls (1.1%), these cases were combined with the CT genotype group to form the “T-allele carrier” group for all statistical tests. The arithmetic means ± standard deviation (SD) was used to express the quantitative data conforming to the normal distribution, and the Student’s test was used to compare the sample mean and the overall mean. Statistical analyses comparing allelic and genotypic distributions were performed using Pearson’s Chi-square test or Fisher’s exact two-tailed test. Moreover, the Hardy-Weinberg equilibrium (HWE) in the patients and the controls was checked using Pearson’s Chi-square test. Power calculations were performed using PS-Power and Sample Size Calculation 3.1.6 software. All the statistical analyses were performed using SPSS 21.0 software for Windows, and the criterion for statistical significance was defined as *P* < 0.05.

## Result

### Association study of SNP (rs7990916) and schizophrenia

There were no significant differences in age or sex between the patient group and controls (*P* = 0.106, *P* = 0.055), indicating the comparability of the data between the two groups (as shown in Table 1). Power analysis indicated that our cohort had the power of 0.999 for identifying a genotype relative risk with an OR of 1.5 at the 0.05 level when we choose a T allele frequency of 0.1524, the finding, therefore, could be statistically strong with appropriate sample size. And the target SNP reaches the HWE both in the patient group and controls (All *P* > 0.05). The distribution of genotype and allele frequencies of linc01080 rs7990916 was shown in Table 2, the frequency of the CC, CT, and TT genotypes was 76.2% (*n* = 868), 21.9% (*n* = 250), and 1.9% (*n* = 21), respectively, in the schizophrenics. And the frequency of these genotypes in the controls was 76.9% (*n* = 799), 22.0% (*n* = 229), and 1.1% (*n* = 11), respectively. Our data revealed that, for the rs7990916 polymorphism, there was no significant difference in the genotype and allele frequency between the patients and the controls (*P* > 0.05). In addition, the analysis of genotypes and allele frequency of rs7990916 in the gender-stratified did not differ between the schizophrenics and controls (shown in Table 3 as follow).

**Table 1:** The demographic characteristics of schizophrenics and controls

Variables	Schizophrenics	Controls	Statistical tests
	*n* = 1139	*n* = 1039	*t* / *χ2 P-value*
Mean Age ± SD (year)	34.44 ± 12.32	33.59 ± 11.74	1.617 *P* = 0.106
Gender n (%)			
Male	722 (63.4%)	617 (59.4%)	3.680 *P* = 0.055
Female	417 (36.6%)	422 (40.6%)	

The enumeration data are presented in the form of mean ± standard deviation

**Table 2:** The genotype and allele frequencies of the *linc01080* gene rs79900916 in the schizophrenics and controls

dbSNP ID	Schizophrenics	Control	*P-value*	OR (95% CI)
	*n* = 1139 (%)	*n* = 1039 (%)		
rs7990916				
Genotype				
CC	868 (76.2)	799 (76.9)	0.314 ^a^	
CT	250 (21.9)	229 (22.0)		
TT	21 (1.9)	11 (1.1)		
CT + TT	271 (23.8)	240 (23.1)	0.703 ^b^	1.030 (0.885–1.199)
Allele				
C	1986 (87.2)	1827 (87.9)		1.000 (reference)
T	292 (12.8)	251 (12.1)	0.461	1.070 (0.894–1.282)

*OR* odds ratio, *95% CI* 95% confidence interval.

^a^Global test for the three different genotypes

^b^Calculations were performed CT + TT vs. CC

**Table 3:** Genotype and allele frequency of the *linc01080* gene rs79900916 in schizophrenics and controls, according to gender

dbSNP ID	Male			Female		
	Patient	Control	*p-value*	Patient	Control	*p-value*
	*n* = 722 (%)	*n* = 617 (%)	OR (95% CI)	*n* = 417 (%)	*n* = 422 (%)	OR (95% CI)
rs7990916						
Genotype						
CC	534 (74.0)	462 (74.9)	0.494^a^	334 (80.1)	337 (79.9)	0.628^a^ *
CT	174 (24.1)	148 (24.0)		76 (18.2)	81 (19.2)	
TT	14 (1.9)	7 (1.1)		7 (1.7)	4 (0.9)	
CT + TT	188 (26.0)	155 (25.1)	0.702^b^	83 (19.9)	85 (20.1)	0.931^b^
Allele						
C	1242 (86.0)	1072 (86.9)	1.000 (reference)	744 (89.2)	755 (89.5)	1.000 (reference)
T	202 (14.0)	162 (13.1)	0.517 1.076 (0.862–1.344)	90 (10.8)	89 (10.5)	0.870 1.026 (0.753–1.399)

*OR* odds ratio, *95% CI* 95% confidence interval.

^a^Global test for the three different genotypes

^b^Calculations were performed CT + TT vs. CC

*Fisher’s exact test

### Association study of SNP (rs7990916) and clinical features of schizophrenics

When it comes to the description of relevant clinical characteristics, no significant differences were observed with regard to duration of illness, Years of education, family history, and PANSS clinical symptom scores between CC group and CT + TT group (All *P > 0.05*). However, we found that there was a difference in the distribution of age of onset in different genotypes, patients carried T allele are at an earlier age of onset than CC genotype carriers (22.10 ± 5.23, 25.14 ± 9.66 respectively) and the difference was extremely marked (*P* < 0.01). (shown in Table 4).

**Table 4:** Clinical characteristics of the patients with schizophrenia and distribution by genotypes of rs7990916

Parameters	rs7990916		*p*-valule
	CC	CT + TT	
	*n* = 868	*n* = 271	
Age at onset (years)	25.14 ± 9.66	22.10 ± 5.23	**8.22E-07**
Years of education (years)	9.60 ± 2.53	9.66 ± 3.05	0.746
PANSS total score	77.64 ± 19.47	76.64 ± 18.30	0.454
P subscore	21.53 ± 7.49	21.89 ± 7.37	0.488
N subscore	18.22 ± 8.71	17.68 ± 8.51	0.371
G subscore	37.89 ± 9.71	37.07 ± 9.74	0.226
Family psychotic history	130 (15.0%)	38 (14.0%)	0.699

Values are the mean ± SD

*PANSS* Positive and Negative Syndrome Scale

### Association study of SNP (rs7990916) and neurocognitive function of schizophrenics

A total of 1039 patients were evaluated for neurocognitive function by BACS (as shown in Table 5). The results showed that the Tower of London scores and the Symbol Coding scores of rs7990916 polymorphism T allele carriers were distinctly lower than those of CC genotype carriers, (for CT + TT group: 6.74 ± 5.68; for CC group: 7.83 ± 6.60), (for CT + TT group: 19.70 ± 13.76; for CC group: 21.83 ± 13.21) and the differences was statistically significant (*P* = 0.014, *P* = 0.022 respectively). Additionally, there was no significant difference in the scores of the other BACS items (including Digit sequencing task, Category instances, Controlled oral word association test, List learning and Token motor task, all *P* > 0.05).

**Table 5:** Neurocognitive functions of the schizophrenics and distribution by genotypes of rs7990916

Parameters	rs7990916		*p-valule*
	CC	CT + TT	
	*n* = 868	*n* = 271	
BACS			
Digit sequencing task	15.02 ± 9.14	15.32 ± 9.64	0.642
Category instances	28.85 ± 12.76	27.35 ± 11.95	0.087
Controlled oral word association test	9.44 ± 5.83	9.02 ± 5.58	0.296
List learning	22.75 ± 14.37	21.22 ± 13.66	0.122
Token motor task	47.63 ± 16.88	49.23 ± 17.31	0.176
Tower of London	7.83 ± 6.60	6.74 ± 5.68	**0.014**
Symbol coding	21.83 ± 13.21	19.70 ± 13.76	**0.022**

Values are the mean ± SD

*BACS* Brief Assessment of Cognition in Schizophrenia

## Discussion

**Fig. 1:**
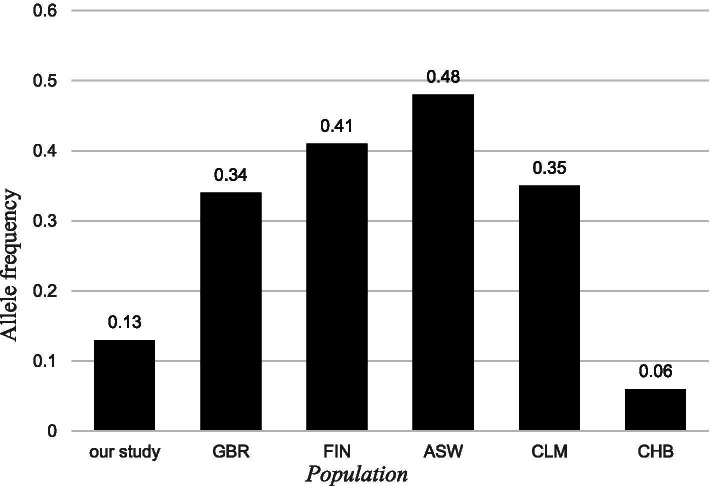
Frequency distribution of the “T” allele of the rs7990916 indifferent ethnic groups. (British in England and Scotland, GBR), (Finish in Finland, FIN),(American of African Ancestry in SW USA, ASW), (Colobians from Medllin, Colobia, CLM) (Han Chinese in Beijing, CHB). (This figure was based on the 1000 Genomes Project)

In this study, we firstly evaluated the potential association between linc01080 rs7990916 polymorphism and schizophrenia. The results showed that there was no significant difference in the frequency distribution of genotypes and alleles between the control group and the case group, and there was still no statistical difference according to the gender stratification study (all *P* > 0.05). However, it is worth noting that the object population of this study is Han people in southern China, and its allele frequency (case group C: T = 0.87:0.13, control group C: T = 0.88:0.12) is different from that of Beijing Han population (C: T = 0.94:0.06), Finnish in Finland (T = 0.041 C = 0.59) and other groups (Fig. 1), which means the racial heterogeneity, so it must need to be researched in different race groups in order to obtain more convincing epidemiological evidence. (based on the 1000 Genomes Project) (https://www.ncbi.nlm.nih.gov/variation/tools/1000genomes/).

Secondly, an interesting phenomenon was found in the analysis of clinical characteristics such as onset age, education level and family history, that is, the onset age of linc01080 rs7990916 T allele carriers was earlier than that of CC genotypes, and the difference was statistically significant (*P* < 0.001). Schizophrenia, as we all know, usually occurs in late adolescence and early adulthood [15], the average age of onset of female patients with schizophrenia is between 25 and 35 years old, while that of male patients is between 18 and 25 years old [16]. Relevant studies have shown that relatively larger gray matter volume defects can be observed in schizophrenia cases with early onset age [17]. The prefrontal cortex is the brain region with the longest duration of ontogeny [18], and puberty marks the final stage of its development [19], which is particularly vulnerable to long-term or repeated stress exposure [20]. A study has shown that hypothalamus-pituitary-adrenal (HPA) axis is activated under stress. Corticosteroids released after activating the HPA axis can not only affect neurogenesis and neuroplasticity, but also high levels of corticosteroids are neurotoxic, which can lead to the degeneration of dendritic processes and the reduction of neuronal survival after injury. Finally resulting in neuronal death [21]. These effects may be characterized by a decrease in the volume of local brain tissue and may lead to the appearance of psychiatric symptoms [22]. In 2012, a study has found that a mutation (linc01080 rs7990916 T > C) had an effect on the volume of gray matter in the brain. It was identified that the gray matter volume of individuals carrying CC was significantly larger than that of individuals carrying TT, and this phenomenon was observed in about 40 brain regions, mainly involving temporal cortex [13], and the gray matter volume of some areas of parietal lobe, frontal lobe and occipital cortex was also affected in the same way [13]. They speculate that the SNP TT genotype may lead to cognitive impairment in patients with Alzheimer’s disease by affecting cortical development in memory-related brain areas (imaging shows reduced gray matter volume) [13]. In fact, these brain regions are not only particularly important for memory function, but also overlap with areas that regulate cognitive functions such as information processing speed and problem solving. In the longitudinal neuroimaging study of patients with first-episode schizophrenia, it was found that the loss of gray matter volume could be observed 2–5 years after onset, especially in the frontal lobe. This phenomenon is more pronounced in adolescents with schizophrenia [23, 24]. Therefore, based on our results, it is suggested that the SNP linc010180 rs7990916 T allele may be associated with the earlier age of onset of schizophrenia by reducing the volume of the frontal cortex, and may also mediate cognitive dysfunction.

There is a view in DSM-IV that cognitive impairment is more severe in patients with schizophrenia who are younger at onset [25]. And some shreds of evidence indicate the earlier the age of onset, the more prominent the clinical symptoms, such as severe negative symptoms and poor execution ability [15]. In a meta-analysis of the age of onset and cognitive function of schizophrenia, it was observed that adolescent patients with schizophrenia had more significant cognitive defects in executive function and psychomotor processing speed than adult patients with first-episode schizophrenia [26]. Therefore, the potential relationship between age of onset and cognitive function cannot be ignored.

Thirdly, through the analysis of the patient’s BACS scale data results, we found that the Symbol Coding and Tower of London scores of rs7990916 T allele in schizophrenics carriers were lower than those of CC genotype carriers, and the differences were statistically significant, indicating T allele aggravate the cognitive function defection. Cognitive dysfunction, considered as the core symptom of psychopathology [27], is prevalent in individuals with schizophrenia. The speed of information processing (tested by Symbol Coding in this study) is one of the most obvious defects in the early stage of schizophrenia [28], it refers to the ability to make the correct corresponding amount in unit time, and is believed to be related to the extensive reduction of gray matter volume and the extensive change of white matter in the frontal and temporal lobes [29]. Although digital symbol coding is rarely the focus of discussion or research, its damage to schizophrenia is significantly greater than the cognitive functions such as verbal memory and executive function that are often studied [30]. In a meta-analysis, information processing speed is even considered to be the center of cognitive impairment in schizophrenia [29].Problem-solving (tested by Tower of London in this study) is an executive function dominated by a neural network centered on the dorsolateral prefrontal cortex (DLPFC) [31]. In related studies on the pathogenesis of schizophrenia, the reduction of DLPFC in the brain has been reported. For example, the expression of the susceptibility gene *COMT* in schizophrenia is related to the size and function of DLPFC [20, 32]. At present, there is extensive evidence that cognitive functions such as executive function, working memory, attention and episodic memory impairment in patients with schizophrenia are related to the volume of the frontal cortex [33]. Therefore, combined with our results, we speculate that the SNP linc010180 rs7990916 T allele may mediate cognitive impairment by reducing the volume of frontal cortex.

In our study, the carriers of the SNP (linc01080 rs7990916) T allele had an earlier age of onset than the CC genotype, and their information processing speed and problem-solving ability were impaired. At the same time, the brain regions responsible for these two cognitive functions were confirmed in previous studies [13] to affect cognitive function through volume reduction. Therefore, we speculate that T allele carriers may be more vulnerable to social pressure and mediate the reduction of the volume of gray matter in the brain, resulting in cognitive impairment in young schizophrenics. However, due to the limitation of technical conditions, whether there is gray matter reduction in corresponding brain regions in schizophrenic patients still needs further observation combined with MRI.

## Conclusion

To sum up, our study found for the first time that the linc01080 rs7990916 T allele may be a risk factor affecting the onset age and cognitive impairment (information processing speed and problem-solving ability) of Han schizophrenic patients in southern China.

## Data Availability

The datasets generated and analyzed during the current study are available in the dbSNP repository, dbSNP accession: {ss2137544294}, (dbSNP Build ID: {B151}). Available from: https://www.ncbi.nlm.nih.gov/SNP/snp_viewTable.cgi?handle=DOP-GDMU-CHINA

## Abbreviations

lincRNA: Long intergenic noncoding RNA
SNP: Single nucleotide polymorphism
imLDR: Improved multiplex ligation detection reaction
BACS: Brief assessment of Cognition
PANSS: Positive and Negative Symptom Scale
HWE: Hardy-Weinberg equilibrium
NSCs: Neural stem cells
HPA: Hypothalamus-pituitary-adrenal
DLPFC: Dorsolateral prefrontal cortex

## References

[1] Lett TA, Chakavarty MM, Felsky D, Brandl EJ, Tiwari AK, Gonçalves VF (2013). The genome-wide supported microRNA-137 variant predicts phenotypic heterogeneity within schizophrenia. Mol Psychiatry.

[2] Brent BK, Thermenos HW, Keshavan MS, Seidman LJ (2013). Gray matter alterations in schizophrenia high-risk youth and early-onset schizophrenia. A review of structural MRI findings. Child Adolesc Psychiatr Clin N Am.

[3] Reif A, Fritzen S, Finger M, Strobel A, Lauer M, Schmitt A (2006). Neural stem cell proliferation is decreased in schizophrenia , but not in depression. Mol Psychiatry.

[4] Hangauer MJ, Vaughn IW, McManus MT (2013). Pervasive transcription of the human genome produces thousands of previously unidentified long intergenic noncoding RNAs. PLoS Genet.

[5] Tuck AC, Natarajan KN, Rice GM, Borawski J, Mohn F, Rankova A (2018). Distinctive features of lincRNA gene expression suggest widespread RNA-independent functions. Life Sci Alliance.

[6] Schmitz SU, Grote P, Herrmann BG (2016). Mechanisms of long noncoding RNA function in development and disease. Cell Mol Life Sci.

[7] Hu J, Xu J, Pang L, Zhao H, Li F, Deng Y (2016). Systematically characterizing dysfunctional long intergenic noncoding RNAs in multiple brain regions of major psychosis. Oncotarget..

[8] Wang C, Li G, Wu Y, Xi J, Kang J (2016). LincRNA1230 inhibits the differentiation of mouse ES cells towards neural progenitors. Sci China Life Sci.

[9] Tang J, Yu B, Li Y, Zhang W, Alvarez AA, Hu B (2019). TGF-β-activated lncRNA LINC00115 is a critical regulator of glioma stem-like cell tumorigenicity. EMBO Rep.

[10] Yu M, Xue Y, Zheng J, Liu X, Yu H, Liu L (2017). Linc00152 promotes malignant progression of glioma stem cells by regulating miR-103a-3p/FEZF1/CDC25A pathway. Mol Cancer.

[11] Chen WL, Chen HJ, Hou GQ, Zhang XH, Ge JW (2019). LINC01198 promotes proliferation and temozolomide resistance in a NEDD4-1-dependent manner, repressing PTEN expression in glioma. Aging (Albany NY).

[12] Zhao Y, Liu H, Zhang Q, Zhang Y (2020). The functions of long non-coding RNAs in neural stem cell proliferation and differentiation. Cell Biosci.

[13] Chen G, Qiu C, Zhang Q, Liu B, Cui Q (2013). Genome-wide analysis of human SNPs at long intergenic noncoding RNAs. Hum Mutat.

[14] Keefe RSE, Harvey PD, Goldberg TE, Gold JM, Walker TM, Kennel C (2008). Norms and standardization of the brief assessment of cognition in schizophrenia (BACS). Schizophr Res.

[15] Esterberg ML, Trotman HD, Holtzman C, Compton MT, Walker EF (2010). The impact of a family history of psychosis on age-at-onset and positive and negative symptoms of schizophrenia: a meta-analysis. Schizophr Res.

[16] Chow TJ, Tee SF, Yong HS, Tang PY (2016). Genetic association of TCF4 and AKT1 gene variants with the age at onset of schizophrenia. Neuropsychobiology..

[17] Torres US, Duran FLS, Schaufelberger MS, Crippa JAS, Louzã MR, Sallet PC (2016). Patterns of regional gray matter loss at different stages of schizophrenia: a multisite, cross-sectional VBM study in first-episode and chronic illness. NeuroImage Clin.

[18] Kaymaz N, van Os J. Heritability of structural brain traits. An endophenotype approach to deconstruct schizophrenia. 1st ed. Int Rev Neurobiol. 2009;89:85–130. Elsevier Inc. Available from: 10.1016/S0074-7742(09)89005-3. 10.1016/S0074-7742(09)89005-3 19900617

[19] Selemon LD, Zecevic N (2015). Schizophrenia: a tale of two critical periods for prefrontal cortical development. Transl Psychiatry.

[20] Cancel A, Comte M, Truillet R, Boukezzi S, Rousseau PF, Zendjidjian XY (2015). Childhood neglect predicts disorganization in schizophrenia through grey matter decrease in dorsolateral prefrontal cortex. Acta Psychiatr Scand.

[21] Sapolsky RM (2003). Stress and plasticity in the limbic system. Neurochem Res.

[22] Valli I, Crossley NA, Day F, Stone J, Tognin S, Mondelli V (2016). HPA-axis function and grey matter volume reductions: imaging the diathesis-stress model in individuals at ultra-high risk of psychosis. Transl Psychiatry.

[23] Van Haren NEM, Hulshoff Pol HE, Schnack HG, Cahn W, Mandl RCW, Collins DL (2007). Focal gray matter changes in schizophrenia across the course of the illness: a 5-year follow-up study. Neuropsychopharmacology..

[24] Frangou S, Hadjulis M, Vourdas A (2008). The Maudsley early onset schizophrenia study: cognitive function over a 4-year follow-up period. Schizophr Bull.

[25] APA (1994). Diagnostic and statistical manual of mental disorders.

[26] Rajji TK, Ismail Z, Mulsant BH (2009). Age at onset and cognition in schizophrenia: Meta-analysis. Br J Psychiatry.

[27] Reichenberg A, Caspi A, Harrington H, Houts R, Keefe RSE, Murray RM (2010). Static and dynamic cognitive deficits in childhood preceding adult schizophrenia: a 30-year study. Am J Psychiatry.

[28] Sakurai T, Gamo NJ, Hikida T, Kim SH, Murai T, Tomoda T (2015). Converging models of schizophrenia - network alterations of prefrontal cortex underlying cognitive impairments. Prog Neurobiol.

[29] Cassetta BD, Goghari VM (2016). Working memory and processing speed training in schizophrenia: study protocol for a randomized controlled trial. Trials.

[30] Dickinson D, Ramsey ME, Gold JM (2007). Overlooking the obvious a meta-analytic comparison of digit symbol coding tasks and other cognitive measures in schizophrenia. Arch Gen Psychiatry.

[31] Ruocco AC, Rodrigo AH, Lam J, Di Domenico SI, Graves B (2014). A problem-solving task specialized for functional neuroimaging: validation of the Scarborough adaptation of the tower of London ( S-TOL ) using near-infrared spectroscopy. Front Hum Neurosci.

[32] Honea R, Verchinski BA, Pezawas L, Kolachana BS, Callicott JH, Mattay VS, Weinberger DRM-LA (2009). Impact of interacting functional variants in COMT on regional gray matter volume in human brain. Neuroimage..

[33] Ehrlich S, Brauns S, Yendiki A, Ho BC, Calhoun V, Charles Schulz S (2012). Associations of cortical thickness and cognition in patients with schizophrenia and healthy controls. Schizophr Bull.

